# Prenatal characteristics and factors contributing to congenital syphilis: A descriptive analysis of cases reported to the Canadian Paediatric Surveillance Program June 2021 through May 2023

**DOI:** 10.17269/s41997-026-01152-7

**Published:** 2026-03-19

**Authors:** Genevieve Gravel, Joanna Merckx, Amandine Bemmo, Kelly Baekyung Choi, Jaskiran Sandhu, Joan Robinson, Ari Bitnun, Jason Brophy, Karen Leis, Laura Sauvé, Sam Wong, Melanie King, Jared Bullard, Carsten Krueger

**Affiliations:** 1 Centre for Communicable Diseases and Infection Control, Public Health Agency of Canada, Ottawa, ON Canada; 2 Department of Pediatrics in the Faculty of Medicine & Dentistry, University of Alberta, Edmonton, AB Canada; 3 Division of Infectious Diseases, Department of Pediatrics, SickKids, University of Toronto, Toronto, ON Canada; 4 CHEO, University of Ottawa, Ottawa, ON Canada; 5 General Pediatrics, Jim Pattison Children’s Hospital, University of Saskatchewan College of Medicine, Saskatoon, SK Canada; 6 Pediatric Infectious Diseases, University of British Columbia, Vancouver, BC Canada; 7 Canadian Paediatric Society, Edmonton, AB Canada; 8 Canadian Paediatric Surveillance Program, Canadian Paediatric Society, Ottawa, ON Canada; 9 Pediatric Infectious Diseases, University of Manitoba, Winnipeg, MB Canada; 10 Section of Pediatric Infectious Diseases, Alberta Children’s Hospital, Calgary, AB Canada

**Keywords:** Congenital syphilis, Prenatal care, Social determinants of health, Risk factors, Substance use, Syphilis congénitale, Prise en charge prénatale, Déterminants sociaux de la santé, Facteurs de risque, Consommation de substances

## Abstract

**Objectives:**

The incidence of congenital syphilis (CS) in Canada increased from 2.1 to 14.5 reported confirmed cases/100,000 live births from 2017 to 2023 (from 8 to 53 cases). We aimed to document prenatal characteristics and contributing factors to CS among mothers or birthing parents (M/BP) of infants with CS in Canada.

**Methods:**

Participants of the Canadian Paediatric Surveillance Program, which includes both paediatricians and paediatric subspecialists, were invited to report on CS cases meeting the study case definition between June 2021 and May 2023. A detailed questionnaire was completed by the reporting clinician. We used descriptive statistics in the assessment of prenatal risk factors among cases, including data on healthcare access, diagnosis, and treatment as well as on socio-demographic, socio-economic and socio-behavioural determinants.

**Results:**

During the 24-month study period, 245 live-born cases of CS were reported, including 81 (33.1%) confirmed and 164 (66.9%) probable cases from seven provinces and territories. Substance use in pregnancy was reported in 65% of cases. Only half of M/BP had at least 1 prenatal care visit during their pregnancy, while only one-quarter had ≥ 1 prenatal care visit in each trimester. In a quarter of cases, no syphilis screening was performed during pregnancy. Prenatal syphilis treatment was not initiated among 20% who screened positive.

**Conclusion:**

In this country-wide assessment, we identified substantial failures in the delivery of adequate prenatal care to M/BP of live-born infants diagnosed with CS. Public health action, such as community outreach to ensure prenatal care for all pregnant people, with specific attention to the prenatal healthcare needs and engagement in the care of those who use substances, is pressing.

**Supplementary Information:**

The online version contains supplementary material available at https://doi.org/10.17269/s41997-026-01152-7.

## Résumé

**Objectifs:**

L'incidence de la syphilis congénitale (SC) au Canada a augmenté de 2,1 à 14,5 cas confirmés déclarés / 100 000 naissances vivantes de 2017 à 2023 (de 8 à 53 cas). Nous avons cherché à documenter les caractéristiques et les facteurs prénataux contribuant à la SC chez les mères ou les parents accoucheurs (M/PA) de nourrissons atteints de SC au Canada.

**Méthodes:**

Les participants au Programme canadien de surveillance pédiatrique, qui comprend des pédiatres et des surspécialistes en pédiatrie, ont été invités à déclarer les cas de SC répondant à la définition du cas d'étude de juin 2021 à mai 2023. Un questionnaire détaillé a été rempli par le clinicien déclarant. Nous avons utilisé des statistiques descriptives pour évaluer les facteurs de risque prénataux parmi les cas, y compris les données sur l'accès aux soins de santé, le diagnostic et le traitement, ainsi que sur les déterminants sociodémographiques, socio-économiques et socio-comportementaux.

**Résultats:**

Au cours de la période d'étude de 24 mois, 245 cas de SC nés vivants ont été signalés, dont 81 (33,1%) cas confirmés et 164 (66,9%) cas probables provenant de sept provinces et territoires. La consommation de substances pendant la grossesse a été signalée dans 65% des cas. Seule la moitié des M/PA ont bénéficié d'au moins une visite de soins prénataux pendant leur grossesse, tandis que seul un quart d'entre elles ont bénéficié de 1 visite de soins prénataux ou plus par trimestre. Dans un quart des cas, aucun dépistage de la syphilis n'a été effectué pendant la grossesse. Le traitement prénatal de la syphilis n'a pas été initié chez 20% des femmes dont le dépistage était positif.

**Conclusion:**

Cette évaluation à l'échelle nationale a mis en évidence des lacunes importantes dans la fourniture de soins prénataux adéquats aux M/PA d'enfants nés vivants chez qui la SC a été diagnostiquée. Il est urgent de prendre des mesures de santé publique, telles que la sensibilisation des communautés pour garantir des soins prénataux à toutes les femmes enceintes, en accordant une attention particulière à l'engagement dans les soins prénataux et aux besoins spécifiques des personnes qui consomment des substances psychoactives.

## Objectives

Congenital syphilis rates have risen dramatically in Canada in recent years. The number of cases of early confirmed congenital syphilis (CS) rose from eight reported nationally in 2017 to 53 in 2023. This corresponds to a rate change from 2.1 to 14.5 per 100,000 live births, an increase of 590% (Public Health Agency of Canada [PHAC],  2025). In contrast, from 1993 to 2004, the number of reported CS cases ranged from one to four cases per year (PHAC, 2020). This increase is a result of the rapidly rising infectious syphilis rates among females of reproductive age. Of note, this is a heterogeneous syphilis epidemic with regionally distinct outbreaks.

The risk of vertical transmission depends on the stage of syphilis infection and gestational age at the time of mother or birthing parent (M/BP) infection and treatment (Fanella et al., 2024). While the probability of adverse outcomes varies across studies, untreated syphilis during pregnancy will lead to adverse outcomes in over half of cases, including miscarriage, stillbirth, prematurity, neonatal death, or CS (Qin et al., 2014). While a substantive number of infected newborns are asymptomatic at birth, they will develop manifestations of late congenital syphilis if not diagnosed and treated (Lopez et al., 2022).

CS is a notifiable disease in Canada through the Canadian Notifiable Disease Surveillance System (CNDSS). However, during the study period, only laboratory-confirmed cases (Supplement 1) from live birth to 2 years of age were reportable. CNDSS collects a limited number of covariates, including province or territory of residence of the M/BP, and sex and age group of the child at the time of diagnosis. Probable CS cases and stillbirths associated with syphilis were not reportable country-wide until January 2024 when a new case definition was introduced (PHAC, 2024a; Supplement 1). Little is known about the prenatal circumstances of women and other M/BPs whose infants are diagnosed with CS in Canada. Syphilis screening is recommended at the first prenatal visit in national guidelines and in all provinces and territories (PHAC, 2024b; Public Health Ontario [PHO], 2023). Some jurisdictions recommend universal additional second and/or third trimester screening and/or testing at delivery, while others suggest doing so in those perceived to be at continued risk of infection (PHAC, 2024b; PHO, 2023). As CS is preventable, its occurrence reveals multiple levels of missed opportunities (McDonald et al., 2023) for timely prenatal screening and treatment.

The objectives of this study were to identify and describe the prenatal characteristics and factors associated with CS in Canada. The focus is on socio-demographic, socio-economic, and socio-behavioural risk factors and determinants; prenatal care access; syphilis testing; and treatment for M/BP of infants with CS.

## Methods

### Study population

Participants of the Canadian Paediatric Surveillance Program (CPSP, a joint project of the Canadian Paediatric Society and the Public Health Agency of Canada), which includes both paediatricians and paediatric subspecialists, were invited each month to report on incident CS cases they encountered in their practice between June 1, 2021, and May 31, 2023. Due to Quebec privacy legislation, cases reported by Quebec participants were excluded from the data analysis. Cases up to the age of 4 years, meeting one of the two study case definitions (i.e., confirmed or probable case of CS, Supplement 1), were reportable to the CPSP on a voluntary basis. Cases reported as probable that had long-bone X-ray changes and/or reactive CSF VDRL were reclassified as confirmed prior to analysis. When reporting a case, clinicians were asked to complete a detailed questionnaire including socio-demographics, healthcare use, prenatal risk factors, and diagnostic and treatment information of both the M/BP and newborn. One of the M/BP variables investigated, entitled “maternal/BP population group”, encompassed race/ethnicity and Indigenous identity, as reported by the respondent. The protocol and detailed questionnaire can be found in Supplement 2. Analysis of the clinical outcomes of the CS cases will be published elsewhere.

### Data analysis

We used descriptive statistics to present total counts, proportions, and means/medians, as appropriate. Where applicable, unknown and missing numbers were combined and used in the denominators such that estimates presented represent a minimum prevalence. Inclusion of any unknown and/or missing data is indicated in the tables, and results for confirmed and probable CS cases are presented together given the small number of cases on stratification. Statistics containing fewer than five data points but more than zero are suppressed and reported as *n* < 5 in the results or merged with another category to preserve confidentiality in accordance with CPSP policy. Consequently, some data are not shown (DNS) to prevent backward calculations. Rural or urban place of residence of the M/BP was determined based on forward sortation area. Rates were estimated per 100,000 live births using Statistics Canada data (Statistics Canada, 2023a). Substance use classes were assessed using the World Health Organization (WHO) Alcohol, Smoking and Substance Involvement Screening Test (ASSIST) principles (Humeniuk et al., 2010). Barriers to prenatal care collected as free-text responses were coded according to available published categories (Supplement 3). We used RStudio software, version 2022.12.0.353, to compute all statistical analyses (R Foundation for Statistical Computing, Vienna, Austria).

## Results

**Fig. 1:**
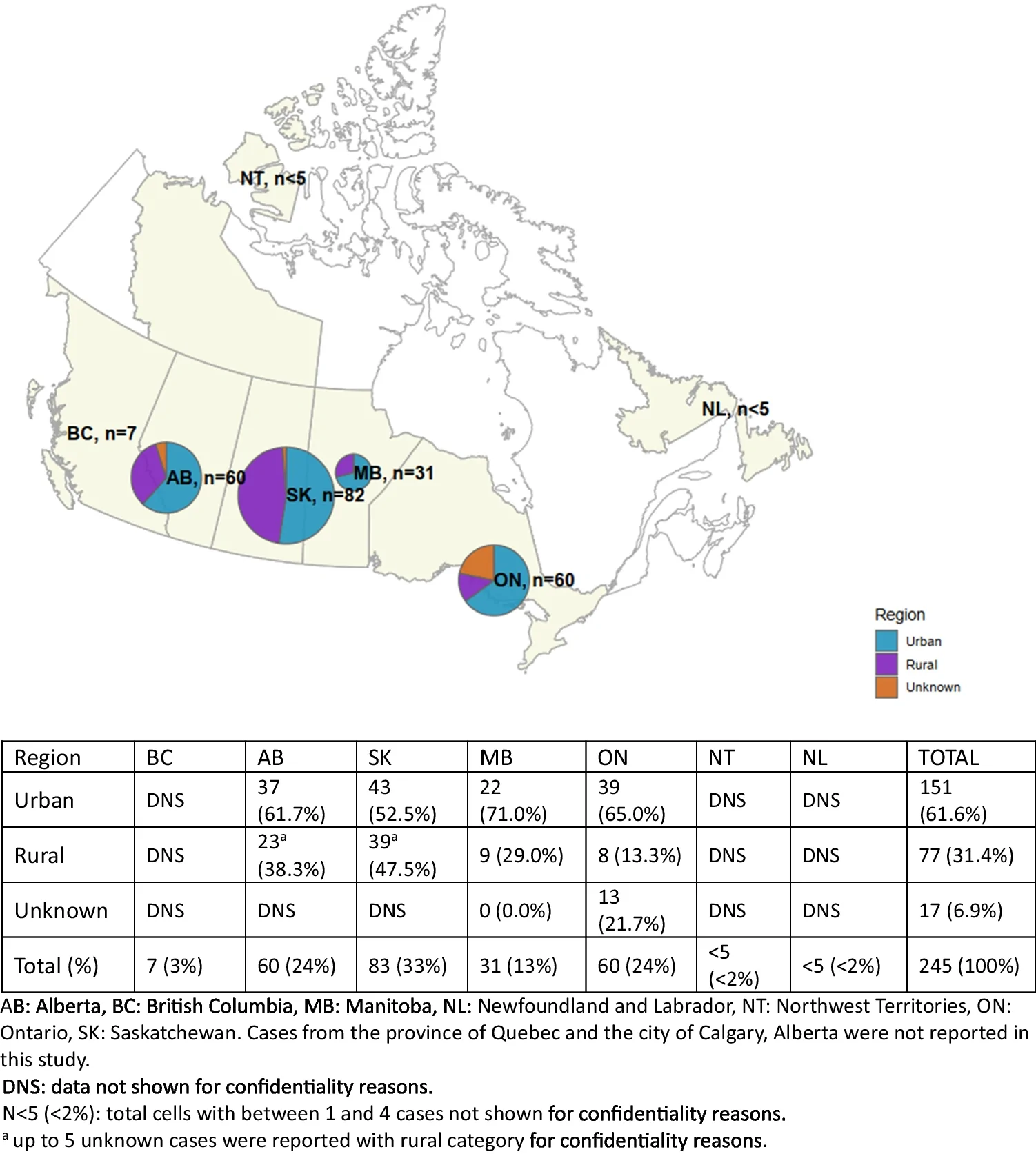
Total number and percentage of reported congenital syphilis cases distribution by geographic location

A total of 245 live-born CS cases were reported. Prior to analysis, 48 cases reported as probable were reclassified as confirmed, resulting in a total of 81 (33.1%) confirmed and 164 (66.9%) probable cases. Cases were reported from six provinces and one territory (British Columbia, Alberta, Saskatchewan, Manitoba, Ontario, Newfoundland and Labrador, and Northwest Territories) (Fig. 1). Most cases (71%; *n* = 173) were reported in the Prairie Provinces (Alberta, Saskatchewan, and Manitoba). All cases were younger than 2 years of age, and most were diagnosed before 2 months of age (97.6%; *n* = 239). The mean M/BP age was 27.2 years (SD 5.6), and 61.6% (*n* = 151) resided in urban regions, with differences by province and territory (Fig. 1). Population group was unknown or missing for a large proportion (46.9%; *n* = 115; including 5 missing). First Nations represent the largest group (38.0%; *n* = 93) overall and in all the participating provinces and territory (data not shown). Respectively, 17.6%, 9.8%, and 26.1% of M/BP were reported as experiencing housing insecurity/homelessness (*n* = 43), receiving social welfare assistance (*n* = 24), and having child protection services involvement with other children (*n* = 64), but data were missing for over half of cases for these variables (Table 1).

**Table 1:** Maternal/birthing parent socio-demographic and socio-economic characteristics in reported congenital syphilis cases

	**Total (*N* = 245)** ***N* (%)**
Age group (years)	
15–19	16 (6.5)
20–24	63 (25.7)
25–29	70 (28.6)
30–39	73 (29.8)
Unknown/missing	23 (9.4)
Population group	
First Nations	93 (38.0)
White	28 (11.4)
Other^a^	9 (3.7)
Unknown/missing (missing = 5)	115 (46.9)
Housing insecurity/homelessness	
Yes	43 (17.6)
No	39 (15.9)
Unknown/missing	163 (66.5)
Social welfare assistance	
Yes	24 (9.8)
No	12 (4.9)
Unknown/missing	209 (85.3)
Child protection services involvement with other children	
Yes	64 (26.1)
No	37 (15.1)
Unknown/missing	144 (58.8)

^a^Other may include cases from Black, Inuit, Latin American, South Asian, or others. Categories reflect the survey question classification which provides categories for race/ethnicity/Indigenous identity, with all in “other” individually counting *N* < 5. Population group is as interpreted by the reporting clinician and not self-reported

Information on substance use during pregnancy was missing for about one-fifth of cases (22.9%; *n* = 56). Substance use was reported in 160 cases (65.3%); of these, 107 (66.9%) reported using substances other than tobacco, alcohol, or cannabis (Table 2), including amphetamine-type stimulants (57.9%; *n* = 62) or opioids (50.5%; *n* = 54). Of those that reported use of substances other than tobacco, alcohol, or cannabis, 38.3% (*n* = 41) used substances from at least two classes, and 8.4% (*n* = 9) used substances from three or more classes (data not shown). Of all M/BP, 18.8% reported intravenous drug use. Data on engagement in sex work/trafficking was reported as unknown or was missing for the majority of the M/BP (80.4%; *n* = 197).

**Table 2:** Maternal/birthing parent socio-behavioural risk factors in reported congenital syphilis cases

	**Total (*N* = 245)** ***N* (%)**
Substance use	
No	29 (11.8)
Yes	160 (65.3)
Missing/unknown	56 (22.9)
Type of substance use	N = 160
Exclusive use of tobacco, alcohol and/or cannabis	27 (16.9)
Other substances with or without tobacco, alcohol and/or cannabis^a^	107 (66.9)
No details provided on the substance used	26 (16.2)
*Detailed type of substance use, other than only tobacco, alcohol, and/or cannabis, information known*^*a,b*^	*n* = *107*
*Opioids*	*54 (50.5)*
*Fentanyl*	*27 (25.2)*
*Amphetamine-type stimulants*	*62 (57.9)*
*Methamphetamines*	*56 (52.3)*
*Crystal meth*	*29 (27.1)*
*Cocaine (coke, crack)*	*34 (31.8)*
*Other (sedatives, hallucinogens, inhalants, etc.)*	*7 (6.5)*
Intravenous drug use	
Yes	46 (18.8)
No	59 (24.1)
Unknown/missing	140 (57.1)
Engaged in sex work/trafficking	
Yes	5 (2.0)
No	43 (17.6)
Unknown/missing	197 (80.4)

^a^May include prescribed substances, e.g., antidepressant, anxiolytic, opioid antagonist, opioids, sedatives

^b^Response options were not mutually exclusive

### Prenatal healthcare utilization, testing, and treatment

Information about prenatal care visit frequency was available for 75.5% of cases (*n* = 185; Table 3). Approximately half had at least one prenatal care visit (50.2%; *n* = 123) with only one-quarter (*n* = 61) having had at least one prenatal care visit in each trimester. For 61 cases (24.9%), the clinician reported suspected barriers to prenatal care including substance use (29.5%; *n* = 18), housing insecurity (23.0%; *n* = 14), patient attitudinal barriers (19.7%; *n* = 12), or structural barriers (16.4%; *n* = 10) (Supplement 3).

**Table 3:** Maternal/birthing parent prenatal care utilization and syphilis stage in reported congenital syphilis cases

	Total (*n* = 245) *N* (%)
At least one prenatal care visit	
Yes	123 (50.2)
No	62 (25.3)
Unknown/missing	60 (24.5)
At least one prenatal care visit in each trimester	
Yes	61 (24.9)
No	124 (50.6)
Unknown/missing	60 (24.5)
Syphilis stage at diagnosis during pregnancy	*n* = 151
Primary	24 (15.9)
Secondary	6 (4.0)
Latent	74 (49.0)
Latent < 12 m	30 (19.9)
Latent > 12 m	19 (12.6)
Latent unknown	25 (16.5)
Unknown/missing^a^	47 (31.1)

^a^Tertiary comprised < 5 cases and was included in this category

**Fig. 2:**
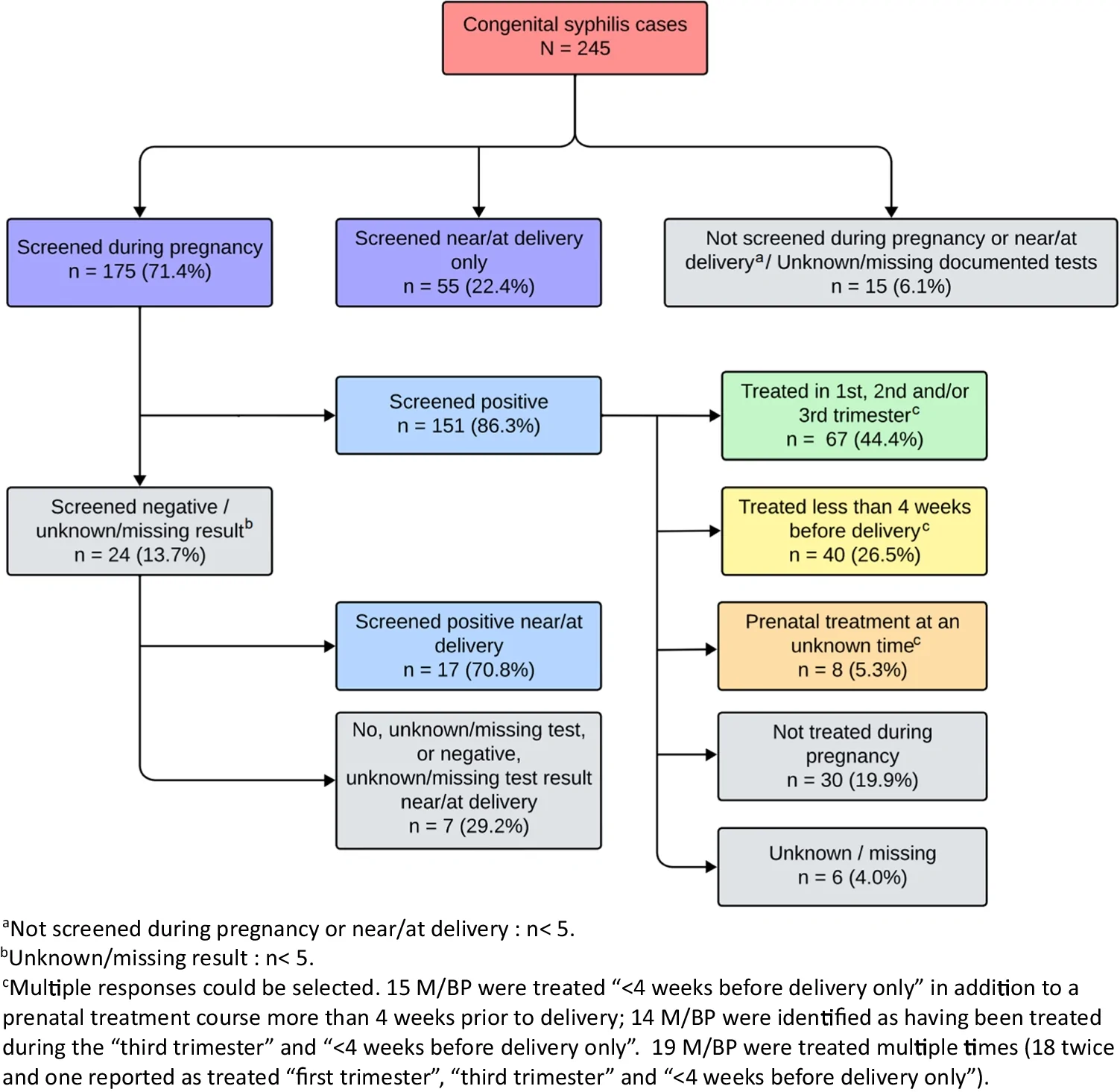
Care cascade among birthing parents of reported congenital syphilis cases

A screening and treatment flow chart is presented in Fig. 2. In 71.4% (*n* = 175) of reported CS cases, the M/BP received syphilis screening during pregnancy, with 151 (86.3%) screening positive during pregnancy. Prenatally diagnosed syphilis was staged as primary, secondary, and early latent syphilis in 15.9% (*n* = 24), 4.0% (*n* = 6), and 19.9% (*n* = 30) of the M/BP, respectively. Latent syphilis of unknown duration was diagnosed in 16.5% (*n* = 25). The stage at prenatal diagnosis was not known or was missing to the reporting clinician in 31.1% (*n* = 47) of the M/BP. A total of 24 M/BP (13.7%) had negative syphilis prenatal screening results or missing/unknown (*n* < 5) prenatal screening results reported. Of 151 M/BP positive on prenatal screening, 20% received no treatment (*n* = 30). Among 115 M/BP who screened positive and received prenatal syphilis treatment, the M/BP received treatment less than 4 weeks before delivery in 34.8% (*n* = 40). Treatment was given in more than one trimester in 16.5% (*n* = 19) of the M/BP; however, the rationale for treatment in different trimesters of pregnancy was unavailable. Non-treponemal tests were positive in 94.7% (*n* = 200) of the 211 M/BP who were tested for syphilis at or near delivery. For 55 M/BP (22.4% of those tested positive peripartum), screening near or at delivery was the only prenatal syphilis testing performed. Among the 24 M/BP who tested negative for syphilis or who had unknown/missing (< 5) test results during pregnancy, 17 (71%) were diagnosed with syphilis at or near delivery.

Almost all M/BP (94.7%) were screened for other sexually transmitted and blood borne infections (STBBI; *n* = 232). One or more maternal co-infections were reported in 39.2% of M/BP of CS cases (*n* = 96): 3.3% (*n* = 8) of all M/BP were positive for HIV, 9.4% (*n* = 23) for hepatitis C virus, and less than 5 cases for hepatitis B virus. Co-infection with chlamydia, gonorrhea, and herpes simplex virus was diagnosed in 24.5% (*n* = 60), 12.7% (*n* = 31), and 2.4% (*n* = 6) of M/BP, respectively.

## Discussion

This descriptive study is only the second contemporary national study on CS in Canada. The present study is the first to use detailed clinical case survey data versus the study by Nelson et al. (2025), which used administrative data, and to which some of the authors of the current paper were co-authors. Our study has yielded many findings of clinical and public health significance. Importantly, in addition to M/BP social determinants of health associated with an outcome of CS in their infant, our study further documented gaps in the care of M/BP of CS cases.

We found that probable cases make up the majority of the cases included in our study. To better understand the burden of CS in Canada, a change in the national case definition with inclusion of probable cases, confirmed late CS, and confirmed and probable syphilitic stillbirth has been enacted since January 2024 (Supplement 1).

Our study confirmed variations in CS rates across Canada, with the Prairie Provinces bearing the highest burden (71% of all cases), consistent with prior reports (Nelson et al., 2025; PHAC, 2023b). In 2022, these provinces accounted for 68% of all confirmed early CS cases nationally reported to CNDSS (PHAC, 2023b). Using the data from this study and the live birth population data, we estimated the rate of CS in the Prairie Provinces to be 129.6 cases per 100,000 live births compared to the national rate of 31.2 per 100,000 live births for 2022.

Our study revealed that 61.7% of M/BP reside in urban areas, a proportion consistent with previous (2017–2020) findings in Manitoba (57.1%) (Tsang et al., 2022) and in Alberta (55.6%) (Gratrix et al., 2022). Heaman et al. (2014) report the following challenges with providing prenatal healthcare in urban environments: transportation, childcare issues, and lack of support. Compared to 2022 vital statistics data in which 16.7% of all Canadian M/BP’s place of residency is rural, in our study, 31.4% of the M/BP who had infants with CS with known place of residence reside in rural settings. In this study, Alberta is the only province where the proportion of M/BP of CS cases with a rural residency was much larger than the provincial proportion of M/BP with rural residency. Overall, 14.2% of all M/BP resided in rural settings in 2022 in Alberta, while 38.3% of the M/BP in Alberta in this study have a rural residence. A previous study from Alberta describes the spread of syphilis into rural and remote areas with limited access to health and social services, contributing to an increase in cases of CS among underserved remote populations (Gratrix et al., 2022). This spread to rural settings is also seen in Manitoba, as demonstrated by positive direct nucleic acid amplification testing rates increasing much more in rural regions versus urban regions between 2017 and 2020 (38.8-fold versus 7.3-fold; Tsang et al., 2022). Similarly, in Quebec, the highest incidence of CS from 2016 to 2021 has been observed in its northern primarily rural region (Blouin et al., 2023). In Ontario, high rates of CS were also observed in some rural and remote health regions (PHO, 2024a). Rates of CS diagnosis among hospital births were higher among cases with rural and remote maternal residence also in Nelson et al. (2025). There are no studies on how mobility within or between provinces and territories and within and between rural and urban regions contributes to the transmission of infectious syphilis. Nonetheless, it appears to be the intensity of heterosexual transmission and high community prevalence in females of reproductive age that have increased the risk of CS occurrence throughout Canada.

M/BP of cases were on average younger (mean = 27.2 years) than the mean age at delivery in Canada in 2022, which was 31.6 years (Statistics Canada, 2023b). Various regional Canadian studies have indicated similar ages for M/BP in CS cases, ranging from medians of 26.5 to 27 years in Manitoba (Benoit et al., 2022), 26.0 to 27.5 years in Alberta (Round et al., 2022; Verghese et al., 2018). In health database records, rates of CS among hospital births were higher in cases with a maternal age of less than 25 years (Nelson et al., 2025).

This study is the first to report Indigenous identity of M/BP of CS cases at the national level. The potentially limited representativeness of the study population, high rates of missing data, and the fact that the maternal/BP population group is reported by the clinician in this study, as well as the general lack of national identity, race, and ethnicity data for M/BP for additional context, make results difficult to interpret. While recognizing the limitations of our analysis, we found that M/BP identified as part of the First Nations population group were disproportionately represented among CS. This finding reinforces findings from several publications from Alberta (Gratrix et al., 2022; Macumber et al., 2022; Round et al., 2022; Verghese et al., 2018). One noted that about 61% of the 117 M/BP were of First Nations self-reported identity from 2017 to 2020 (Gratrix et al., 2022). In another, participants who self-identified as Indigenous experienced longer time delays to access prenatal care compared to M/BP who did not identify as Indigenous (Round et al., 2022). It has been well documented that systemic racism, ongoing colonialism, and the effects of intergenerational trauma directly lead to health inequity for Indigenous peoples (PHAC, 2020). It is important to note that the increased risk of infectious syphilis and CS in First Nations M/BP is not inherently due to their Indigeneity; rather, it is a failure of our health and social systems to adequately address health inequity, racism, and colonialism. As such, any intervention to reduce CS must address the structural and social determinants of health that may contribute to the overrepresentation of First Nations M/BP among infectious syphilis and CS.

In our study, 65.3% of M/BP of CS cases reportedly used substances and 18.8% reportedly used injection drugs, consistent with associations between substance use and vertical transmission of syphilis previously reported in Alberta, British Columbia, Quebec, Manitoba, and Ontario (Macumber et al., 2022; Vijh et al., 2022; Blouin et al., 2023; Benoit et al., 2022; Gratrix et al., 2022; PHO, 2024b; Nelson et al., 2025). A cohort study of pregnant people treated for infectious syphilis in late pregnancy in Alberta from 2015 to 2020 found a prevalence of substance use of 59.5% (Macumber et al., 2022). In British Columbia, analysis of all maternal syphilis cases from July 2018 to September 2022 revealed that 73.6% of M/BP for whom information was available reported substance use (Vijh et al., 2022). Additionally, prenatal injection drug use was reported in 21.0% of pregnant people diagnosed with syphilis in Alberta from 2017 to 2020 (Gratrix et al., 2022). In Ontario, a retrospective case review of CS cases from 2020 to 2022 found that 58.3% of the M/BP reported substance use and 52.8% reported being homeless/underhoused (PHO, 2024b). In Nelson et al. (2025), the rate ratio of exposure to substances of addiction in utero among infants with a CS compared to infants without was 27.9 (95% CI 24.8–31.3), and the authors stated this factor may be underreported. Further investigations are needed to understand the association between substance use and CS outcomes. Canada is experiencing a syndemic where syphilis and substance use adversely interact and are made more deleterious by experienced inequities such as housing insecurity/homelessness (The Lancet, 2017). Stimulants, particularly the use of methamphetamine, are known to enhance sexual behaviours that augment the risk of syphilis infection (Strathdee et al., 2021). Other substances, like opioids, and opioid use disorder have been linked to the exchange of sex for money or sex for substances (Strathdee et al., 2021). Additionally, people who use drugs are more likely to experience stigma and to express mistrust of the healthcare system (Singh & Romanowski, 2019). Associations between substance use and inadequate prenatal care have been documented in Canadian studies (Nussey et al., 2020; Round et al., 2022). On the other hand, challenges accessing prenatal care associated with unstable/inadequate housing reported by public health unit staff in Ontario included healthcare providers struggling to contact clients for appointments or follow-ups (e.g., phone number out of service, wrong address) and clients losing their health cards needed for provincially funded health services (PHO, 2024b). Other challenges accessing prenatal care mentioned were transportation challenges, pregnancy awareness, and health systems issues. Fear of child apprehension is directly cited as a reason for disengagement from, avoidance of, or delayed presentation to prenatal care services in the Canadian literature (Wall-Wieler et al., 2019).

We have identified gaps in the care of M/BP of CS cases, revealing missed opportunities for prevention. In our study, only half of the M/BP had at least one prenatal care visit during their pregnancy, a WHO standard for prenatal care, while only a quarter had at least one prenatal care visit in each trimester. One-quarter were not screened for syphilis during pregnancy. And even among those who screened positive, one-fifth of M/BP had not initiated treatment, demonstrating that expanded testing must be accompanied by efforts to facilitate adequate treatment in order to avoid missed opportunities. In contrast, 76.2% of M/BP screened positive during pregnancy received treatment. This contrast emphasizes the crucial role of ‘timely’ testing in facilitating treatment initiation. Regional surveillance studies have documented absent or delayed prenatal care in 50%, 71.4%, and 72.2% of M/BP of CS cases, respectively (Benoit et al., 2022; Round et al., 2022; PHO, 2024b). Surveillance data also showed that even when M/BP have access to prenatal care, they may not necessarily receive timely screening for syphilis during their pregnancy, nor timely or adequate treatment for syphilis after screening positive (Benoit et al., 2022; Blouin et al., 2021, 2023; Round et al., 2022; PHO, 2024b). In Manitoba (2012–2018), 44.4% of the 9 M/BP of cases of CS were tested for the first time and diagnosed with syphilis during delivery or postpartum (Lopez et al., 2022). In Alberta, in 2017 through 2019, 63.5% of 63 M/BP of infants with CS had their first syphilis screening performed in the third trimester or peripartum, and a quarter of the M/BP had an interval of over 48 days between their screening and receiving treatment (Round et al., 2022). Importantly, it has been shown that late syphilis screening during pregnancy (third trimester or less than 28 days before delivery) (Gratrix et al., 2022) and later treatment initiation (Round et al., 2022) are significantly and independently associated with an outcome of CS. These findings suggest the need for strategies to reduce barriers to syphilis diagnosis, management, and care, given the significant recent increases in CS in Canada. The complementary use of point-of-care testing by healthcare teams, enabling immediate treatment and seamless integration into medical care, is one example.

The importance of repeated prenatal syphilis screening has been previously demonstrated for detecting individuals who seroconvert after the first-trimester screening, as is the case when individuals are tested in the window period or when there is a new or re-infection during pregnancy. Studies from Alberta, Manitoba, Quebec, British Columbia, and Ontario have reported several cases of CS where the M/BP initially tested negative for syphilis during early prenatal screening but screened positive near or at delivery, or post-delivery (Benoit et al., 2022; Blouin et al., 2021, 2023; Gratrix et al., 2022; Lopez et al., 2022; Round et al., 2022; Vijh et al., 2022; PHO, 2024b). Affirming these findings, we found that among the one-sixth of M/BP of cases who had tested negative for syphilis during pregnancy, 81% were at least identified with syphilis at or near delivery. Although it is unclear when infection occurred for these M/BP, repeat testing in pregnancy likely would have allowed treatment prior to birth and potentially prevented infection of the infant.

While HIV co-infections are routinely reported among pregnant syphilis cases in Canada, no co-infections among M/BP were reported in other Canadian studies analyzing data from 2017 to 2020 (PHO, 2024b, Gratrix et al., 2022; Vijh et al., 2022; Willemsma et al., 2022). The 3.3% of HIV co-infection among M/BP of CS cases found in our sample is disproportionately higher than the national percentage of live births to HIV positive M/BP in 2022 of 0.065% (PHAC, 2023a). A high proportion of hepatitis C virus co-infection was also found (9.4%). This is, however, three times lower than the 29.4% co-infection proportion reported among maternal syphilis cases in Ontario (PHO, 2024b). Unfortunately, data to compare co-infections with other studies are limited.

This study has several limitations. The study population does not include all CS cases in Canada during the study period and may not be nationally representative, primarily because the study relied on voluntary reporting by clinicians and not all provinces and territories participated. The COVID-19 pandemic may also have contributed to underreporting due to other competing priorities for reporting clinicians. The case definition used in this study is more sensitive than the national case definition in effect during the study period, with all infants treated for CS eligible (Supplement 1). Data missingness on some of the variables did not allow us to verify the reported classification (i.e., probable versus confirmed) in all cases. In addition, stillbirths, a severe and common outcome of antepartum syphilis transmission, were not included in this study. In terms of documentation of risk factors and determinants, there may be a strong social desirability bias against reporting risk behaviour to the clinician, but conversely, clinicians may also need to improve patient data gathering in view of optimizing support for individual patients. Many variables were unknown to reporting clinicians. Missingness of sensitive variables is large, and the reliable collection of socio-demographic information remains challenging even in enhanced surveillance studies. Some data elements regarding M/BP prenatal care, screening, and treatment appear to not have been available in the records of CS cases at the time of reporting and are therefore missing. The questionnaire did not allow us to determine the trimester of M/BP syphilis screening or the temporal relationship between screening and (re)infection. An additional limitation is that as information was collected without any comparator group, we cannot estimate the absolute risks related to the described variables. Our data thus cannot estimate how many cases could be averted by potential prenatal care interventions. Some variables that have been previously identified as risk factors or determinants were not assessed in this study. Lastly, the total sample size remains small, limiting group comparisons and further analysis.

Further qualitative research is needed to understand socio-demographic, systemic, and structural factors that create barriers to accessing comprehensive prenatal care for pregnant people at risk of syphilis. Information on prenatal care systems and structures linked to our study population, including the organization and delivery of prenatal and postnatal care, could provide insight into barriers but also facilitators experienced by M/BP during pregnancy. Barriers to accessing prenatal care and to syphilis treatment initiation and completion need further attention. This should focus on ensuring culturally safe, First Nations-led where appropriate, and non-stigmatizing approaches, as well as broader women’s health rights issues, particularly primary prevention of both syphilis and unwanted pregnancy. These results, together with continued surveillance, can inform future research as well as policy and programming responses to the current outbreak.

## Conclusion

In Canada, the number of CS cases has reached the highest level in decades. The inclusion of probable cases in the reporting of cases is essential to a more representative reflection of the burden of disease. These findings support the recent expansion of the national case definition. This analysis identified failures in delivery of prenatal services in general, and syphilis diagnosis and treatment in particular, to birthing parents of CS cases. Our data indicate that infectious and CS, substance use, homelessness or housing instability, and possibly HIV exist as a syndemic in Canada, with regional heterogeneity. Research to better understand and interventions to address the syndemic, including identifying and mitigating barriers to healthcare with specific attention to the prenatal healthcare needs of those who use substances, are needed.

## Requirement for meaningful engagement of First Nations, Inuit, Métis, and Indigenous Peoples in publications about them

1. *Are First Nations, Inuit, Métis, and/or Indigenous Peoples or populations a focus of this submission?* First Nations, Métis, and Indigenous Peoples are not the focus of this publication. However, individuals with First Nations’ identity, as reported by the treating clinician and specified in the manuscript, constitute a meaningful proportion (38%) of the mothers/birthing parents (M/BP) of the cases reported upon in this manuscript. The collection of race/ethnicity/identity data was part of the REB-approved study protocol.
2. *If yes, were the relevant Indigenous Peoples or populations engaged in the study and/or preparation of this submission?*
  Indigenous Peoples or populations were unfortunately not engaged in the preparation of this study for various reasons including the fact that at the time the study was launched, besides the REB approvals of all variables to be collected, there were no Diversity, Equity, and Inclusion (DEI) guidelines nor specific practices with regards to community engagement in place for studies performed through the Canadian Paediatric Surveillance Program of the Canadian Paediatric Society (CPS). They were however engaged in the preparation of this submission, see details below.
3. *If yes, please summarize how the relevant Indigenous Peoples or populations have been engaged as individuals and collectively in the study and/or preparation of this submission (2000 character limit).*
  We pro-actively, during the preparation of the manuscript, engaged with representatives of the Assembly of First Nations and of the First Nations Information Governance Centre, two national bodies representing First Nations, to present this finding and the final draft of the manuscript before submission was shared with them for review.
  Additionally, after the initial submission of our manuscript, we reached out to CPS to enquire as to whether they now had DEI measures in place with regards to work affecting Indigenous Peoples or populations. We were informed that they have a First Nations, Inuit and Metis Health Committee in place. The co-chairs of this group have reviewed and provided comments on the current version of the manuscript which we addressed and included.
  The proportion of M/BP of First Nations’ identity was already published in the 2023 CPSP annual report with the ok from AFN representative.
  The finding is contextualized in the manuscript discussion to prevent stigmatization of First Nations populations.
  We decided not to include the increased burden of disease by race/ethnicity/identity in the abstract to ensure the readers would only be provided this specific datapoint while reading the contextual information.
  Limitations, including how Indigenous identity was collected, are further discussed in the paper to help readers understand the boundaries and applicability of the findings.

## Contributions to knowledge

What does this study add to existing knowledge?

- This is the second national study published in recent years on congenital syphilis in Canada; however, it is the first to use detailed clinical case survey data. The previous study by Nelson et al. (2025), to which some of the authors of the current paper were co-authors, used discharge diagnoses from an administrative database for hospital births, with limited individual-level details.
- Our study demonstrates the frequent occurrence of missed congenital syphilis prevention opportunities, including failure or delays in maternal screening and treatment at the national scale.
- Our study reaffirms the importance of substance use, homelessness or housing instability, and rurality as risk factors for congenital syphilis identified in Nelson et al. (2025) and other regional studies.

What are the key implications for public health intervention, practices, or policy?

- The importance of repeated screening in a context of high re-infection rates, especially among people who may become pregnant or are pregnant, to prevent congenital syphilis.
- The need for research and interventions to improve access to care for all and specifically for pregnant people already facing additional barriers and inequities.
- The need for outreach services that address the syndemic conditions to ensure people in need are met “where they are at.”

## Supplementary Information

Below is the link to the electronic supplementary material.ESM1(DOCX 549 KB)ESM2(DOCX 70.6 KB)

## Data Availability

Data are not available following the Canadian Paediatric Surveillance Program rules.

## Code availability

R codes syntax files are not available for request.
