# Supplementary material for: Prenatal characteristics and factors contributing to congenital syphilis: A descriptive analysis of cases reported to the Canadian Paediatric Surveillance Program June 2021 through May 2023
Source: Can J Public Health. 2026 Mar 19;117(4):795–806. doi: 10.17269/s41997-026-01152-7 (PMC13534403; doi:10.17269/s41997-026-01152-7)
Supplement: Supplementary file 1 [file 41997_2026_1152_MOESM1_ESM.docx]

**Figures**

**Figure 1. Total number and percentage of reported congenital syphilis cases distribution by geographic location**

| Region | BC | AB | SK | MB | ON | NT | NL | TOTAL |
| --- | --- | --- | --- | --- | --- | --- | --- | --- |
| Urban | DNS | 37 (61.7%) | 43 (52.5%) | 22 (71.0%) | 39 (65.0%) | DNS | DNS | 151 (61.6%) |
| Rural | DNS | 23^a^ (38.3%) | 39^a^ (47.5%) | 9 (29.0%) | 8 (13.3%) | DNS | DNS | 77 (31.4%) |
| Unknown | DNS | DNS | DNS | 0 (0.0%) | 13 (21.7%) | DNS | DNS | 17 (6.9%) |
| Total (%) | 7 (3%) | 60 (24%) | 83 (33%) | 31 (13%) | 60 (24%) | <5 (<2%) | <5 (<2%) | 245 (100%) |

AB: Alberta, BC: British Columbia, MB: Manitoba, NL: Newfoundland and Labrador, NT: Northwest Territories, ON: Ontario, SK: Saskatchewan. Cases from the province of Quebec and the city of Calgary, Alberta were not reported in this study.

DNS: data not shown for confidentiality reasons.

N<5 (<2%): total cells with between 1 and 4 cases not shown for confidentiality reasons.

^a^ up to 5 unknown cases were reported with rural category for confidentiality reasons.

**Figure 2. Care cascade among birthing parents of reported congenital syphilis cases**

^a^Not screened during pregnancy or near/at delivery : n< 5.

^b^Unknown/missing result : n< 5.

^c^Multiple responses could be selected. 15 M/BP were treated “<4 weeks before delivery only” in addition to a prenatal treatment course more than 4 weeks prior to delivery; 14 M/BP were identified as having been treated during the “third trimester” and “<4 weeks before delivery only”. 19 M/BP were treated multiple times (18 twice and one reported as treated “first trimester”, “third trimester” and “<4 weeks before delivery only”).
