# Supplementary material for: Prenatal characteristics and factors contributing to congenital syphilis: A descriptive analysis of cases reported to the Canadian Paediatric Surveillance Program June 2021 through May 2023
Source: Can J Public Health. 2026 Mar 19;117(4):795–806. doi: 10.17269/s41997-026-01152-7 (PMC13534403; doi:10.17269/s41997-026-01152-7)
Supplement: Supplementary file 2 [file 41997_2026_1152_MOESM2_ESM.docx]

**Prenatal characteristics and factors contributing to congenital syphilis: A descriptive analysis of cases reported to the Canadian Paediatric Surveillance Program June 2021 through May 2023**

**Supplements**

**Supplement 1: Study case definitions congenital syphilis cases and national prior and current case definitions**

| **Study case definitions** | |
| --- | --- |
| **Confirmed case** | Liveborn (within 4 years of age)  Identification of *Treponema pallidum* in any specimen type of the infant or child by   - polymerase chain reaction (PCR) or - fluorescent antibody examination, or - reactive serology from a venous blood sample of the infant measuring a four-fold higher titer compared to birthing parent collected near the time of birth, or   persistent reactive serology in a child beyond the second birthday |
| **Probable case** | Liveborn (within 4 years of age)   - Infant born to a birthing parent who had untreated or inadequately treatment at delivery regardless of clinical findings in the newborn, or - an infant with a reactive treponemal tests and one of the following:   i) clinical signs or symptoms of congenital syphilis,  ii) evidence of syphilis infection related lesions on radiographs of the long bones;  iii) abnormal cerebrospinal fluid (CNS) white blood cell (WBC) count or increased protein level without an alternate cause;  iv) reactive treponemal immunoglobin M. |
| **National case definition in effect during the study period** | |
| **Confirmed early congenital syphilis** | Laboratory confirmation of infection (within 2 years of birth):   - identification of *Treponema pallidum* by dark-field microscopy, fluorescent antibody or equivalent examination of material from nasal discharges, skin lesions, placenta, umbilical cord or autopsy material of a neonate (up to four weeks of age)   or   - reactive serology (non-treponemal and treponemal) from venous blood (not cord blood) in an infant/child with clinical, laboratory or radiographic evidence of congenital syphilis^1^ whose mother is without documented evidence of adequate treatment   or   - detection of *T. pallidum* DNA in an appropriate clinical specimen |

| **National case definition in effect from January 2024**^2^ | |
| --- | --- |
| **Confirmed case: Early congenital syphilis** | Laboratory confirmation of infection in a live birth:   - identification of *Treponema pallidum* by nucleic acid detection (PCR or equivalent), fluorescent antibody or equivalent examination of material in an appropriate clinical specimen (see Laboratory comments)   **or**   - reactive serology (non-treponemal **and** treponemal) from venous blood (not cord blood) in an infant or in a child **with** clinical, radiographic or other laboratory evidence of congenital syphilis^3^   **or**   - infant's RPR titre at least fourfold higher than the mother/birthing parent's RPR titre in samples collected during the immediate postnatal period   **or**   - persistent positive treponemal serology in a child older than 18 months of age   **and**   - younger than two years of age at the time of meeting the criteria **and** no other suspected source of exposure |
| **Confirmed case: Late congenital syphilis** | Laboratory confirmation of infection:   - identification of *Treponema pallidum* by nucleic acid detection (PCR or equivalent), fluorescent antibody or equivalent examination of material in an appropriate clinical specimen (see Laboratory comments)   **or**   - reactive serology (non-treponemal **and/or** treponemal) in an individual **with** clinical, radiographic or other laboratory evidence of congenital syphilis^3^   **and**   - two or more years of age at the time of meeting the criteria **and** no other suspected source of exposure |
| **Probable case: Early congenital syphilis** | Does not meet criteria for "Confirmed case: Early congenital syphilis"  **and**   - reactive serology (non-treponemal **and/or** treponemal) from venous blood (not umbilical cord blood) in an infant or in a child whose mother/birthing parent had untreated or inadequately treated^4^ syphilis prior to delivery   **and**   - younger than two years of age at the time of meeting the criteria **and** no other suspected source of exposure |
| **Confirmed case: Syphilitic stillbirth** | A fetal death that occurs after 20 weeks' gestation or in which the fetal weight is greater than 500 g with laboratory confirmation of infection [i.e., identification of *Treponema pallidum* by nucleic acid detection (PCR or equivalent), fluorescent antibody or equivalent examination of material in an appropriate clinical specimen (see Laboratory comments)] |

| **Probable case: Syphilitic stillbirth** | Does not meet criteria for "Confirmed case: Syphilitic stillbirth"  **and**   - a fetal death that occurs after 20 weeks' gestation or in which the fetal weight is greater than 500 g where the mother/birthing parent had untreated or inadequately treated^4^ syphilis prior to delivery   **and**   - no other cause of stillbirth established |
| --- | --- |

^1^ Includes any evidence of congenital syphilis on physical examination (e.g. hepatosplenomegaly), evidence of congenital syphilis on radiographs of long bones, a reactive CSF VDRL, an elevated CSF cell count or protein without other cause

^2^ Public Health Agency of Canada. (2024a). National case definition: Congenital Syphilis. Government of Canada. https://www.canada.ca/en/public-health/services/diseases/syphilis/health-professionals/national-case-definition-congenital-syphilis.html

^3^ Includes any evidence of congenital syphilis such as any features suggestive of congenital syphilis on radiographs of long bones; reactive cerebrospinal fluid (CSF) venereal disease research laboratory (VDRL); an elevated CSF cell count or protein (without other cause); anemia; skeletal abnormalities (e.g., osteochondritis, saber shins); hepatosplenomegaly; skin rash; condylomata lata; rhinitis (snuffles); pseudoparalysis; meningitis; ascites; interstitial keratitis; lymphadenopathy; dental abnormalities (e.g., Hutchinson's teeth, mulberry molars); sensory neural hearing loss; intrauterine growth restriction; prematurity; or any other abnormality not better explained by an alternative diagnosis.

^4^ Adequate treatment is:

- treatment with penicillin therapy appropriate for the stage of syphilis infection that was completed at least 4 weeks before delivery; and
- sufficient reduction in maternal/birthing parent non-treponemal titres; and
- no evidence of reinfection.

A lack of verbal or written confirmation of treatment should be considered "inadequate treatment." Refer to current Canadian guidelines for additional information.

**Supplement 2: Canadian Paediatric Surveillance Program Congenital syphilis study protocol and detailed questionnaire**

**Source:** Canadian Paediatric Surveillance Program. (2024). *Congenital Syphilis*. Canadian Paediatric Surveillance Program. https://cpsp.cps.ca/surveillance/study-etude/congenital-syphilis

**Protocol:**

Congenital syphilis

**Principal investigators**

Carsten Krueger, MD, University of Ottawa, Division of Infectious Diseases; ckrueger@cheo.on.ca

Jared Bullard, MD, University of Manitoba, Division of Pediatric Infectious Diseases; jared.bullard@gov.mb.ca

**Co-investigators**

Ari Bitnun, MD, University of Toronto, Division of Paediatric Infectious Diseases

Jason Brophy, MD, University of Ottawa, Division of Paediatric Infectious Diseases

Jeannette Comeau, MD, MSc, Dalhousie University, Division of Infectious Diseases

Joanne Embree, MD, University of Manitoba, Division of Infectious Diseases and Medical Microbiology

Sindu Govindapillai, MD, University of Ottawa, Qikiqtani General Hospital

Michael Issac, MD, University of Manitoba, Medical Officer of Health

David Johnson, MD, University of Calgary, Department of Pediatrics

Karen Leis, MD, University of Saskatchewan, Division of General Pediatrics

Joan Robinson, MD, University of Alberta, Division of Infectious Diseases

Brent Roussin, MD, University of Manitoba, Chief Public Health Officer

Laura Sauvé, MD MPH, University of British Columbia, Division of Infectious Diseases

Holden Sheffield, MD, University of Ottawa, Pediatrics Lead, Qikiqtani General Hospital

Susanna Talarico, MD, University of Toronto, Division of General Paediatrics

Kristina Tomas, MSc, Public Health Agency of Canada

Sam Wong, MD, Northwest Territories Health and Social Services Authority - Stanton Territorial Hospital

**Background**

Syphilis is the third most common sexually transmitted bacterial infection in Canada, with a dramatic and rapid increase in confirmed cases over recent years, on top of a steady increase over the last decade (1,2). Although the majority of these cases are among men, cases among young women are of public health importance due to the potential for vertical transmission to the fetus or neonate during pregnancy. Vertical transmission can occur at any stage of pregnancy and can vary by the stage of illness in the mother, but it is estimated that between 60 to 100% of infants born to untreated mothers with primary or secondary stage infections will become infected (3). While antibiotic treatment in the neonatal period can prevent late manifestations of congenital syphilis, initiation of treatment in the infants of pregnant women is challenging in circumstances where prenatal care is limited and syphilis screening tests were never completed prenatally.

Paralleling the increase in syphilis among Canadian adults, the Public Health Agency of Canada has detected an increase in the incidence of congenital syphilis. In the decade prior to 2005, approximately two cases per year of congenital syphilis were reported nationally. From 2005 to 2017, an average of five cases per year was reported (1,4). In 2018, 17 cases of congenital syphilis were reported nationally, the highest incidence in 25 years. In Manitoba alone, over 26 confirmed cases of congenital syphilis were reported from 2015 to 2019 inclusive (5). Additionally, over 80 syphilis-exposed infants were reported in the first nine months of 2019 alone (5).

In those who survive the prenatal period, congenital syphilis infection impacts multiple organ systems over a lifetime. In-utero manifestations of congenital infection include stillbirth, hydrops fetalis, and preterm birth. Neonatal manifestations, when present, include hepatosplenomegaly, lymphadenopathy, rash, copious secretions (“snuffles”), osteochondritis, hemolytic anemia, thrombocytopenia, and pneumonia (3). Late manifestations, which are preventable by early treatment, include sensory deficits, musculoskeletal deformities, and developmental impairment (3).

The disease can prove challenging to diagnose at birth, as about 60% of infected neonates are asymptomatic (6). Moreover, there is confusion among clinicians regarding the interpretation of maternal and infant syphilis serologies, a problem compounded by both false negatives and false positives in late pregnancy. Taken together, these two challenges likely result in under-recognition of cases of congenital syphilis and it is likely that cases of “probable” congenital syphilis are not included in incidence figures reported by the Public Health Agency of Canada.

All provinces and territories voluntarily submit limited data on nationally notifiable diseases to the Canadian Notifiable Disease Surveillance System (4). While congenital syphilis is nationally reportable, case-level data on the diagnosis, treatment, and ongoing management of congenital syphilis in Canada is lacking. The American Academy of Pediatrics’ 2018 *Red Book* recommends that neonates born to women testing positive for syphilis undergo hematologic, cerebrospinal fluid, and radiographic investigations (testing depends on case specifics); however, it is unclear to what degree these investigations are completed in each case (3). The appropriateness, completeness of, and results from this work up are subsequently used to diagnose congenital syphilis, and to determine the duration of antibiotic therapy (either a single dose of intramuscular benzathine penicillin G or 10 days of intravenous aqueous penicillin G). Many affected neonates are unknowingly treated for inadequately short durations, or the diagnosis is missed all together.

**Methods**

Through the standard methods of the Canadian Paediatric Surveillance Program (CPSP), approximately 2,800 paediatricians and paediatric subspecialists will be actively surveyed on a monthly basis to determine whether they have seen a new confirmed or suspected case of congenital syphilis in the preceding month. Respondents who have seen a case will receive a detailed questionnaire to complete in order to gather case-level information on demographics, diagnostic evaluation, treatment and follow up, and referrals.

The study investigators include representatives from a variety of subspecialties such as infectious diseases, public health, medical microbiology, and general paediatrics from across the country. The investigators will use their national connections to promote reporting of cases in subspecialty circles, at their institutions, and through regional platforms.

### Case definition

Report any neonate, infant, or child <4 years old with a new diagnosis of confirmed or probable congenital syphilis in the last month.

**Confirmed congenital syphilis (requires one of the following)**

1. Identification of *Treponema pallidum* in the infant/child’s specimen by polymerase chain reaction (PCR) or fluorescent antibody examination
2. Reactive serology from venous blood in an infant that is four-fold greater than the maternal serology collected near the time of birth
3. Reactive serology from venous blood in an infant that persists beyond their second birthday

**Probable congenital syphilis**

1.	Infant born to a mother who had untreated or inadequately treated syphilis at delivery, regardless of findings in the infant

OR BOTH OF THE FOLLOWING:

2.	An infant or child with a reactive treponemal test result

3.	One of the following additional criteria:

a.	Clinical signs of congenital syphilis on physical examination

b.	Evidence of congenital syphilis on radiographs of long bones

c.	Abnormal cerebrospinal fluid cell count or protein without other cause

d.	Reactive treponemal immunoglobulin M (IgM) (19S-IgM antibody test or IgM enzyme-linked immunosorbent assay)

Objectives

1) Describe the minimum incidence and national distribution of new cases of confirmed or probable congenital syphilis
2) Identify common risk factors of mothers with affected children and the treatment they received during pregnancy
3) Describe diagnostic testing, management, and complications of neonates, infants and children with confirmed or probable congenital syphilis

Duration

***TBD***

Expected number of cases

Congenital syphilis is a reportable condition with 17 confirmed cases nationally in 2018 according to the Public Health Agency of Canada. It is difficult to estimate the number of cases that will be reported through the CPSP. Not all cases of congenital syphilis will be seen by paediatricians and subspecialists participating in the CPSP reporting system and many neonates and infants likely fall under the umbrella of “probable congenital syphilis” and were not captured in national reporting figures. Based on these factors, no more than 250 cases are expected over a two-year period.

### Study limitations

Congenital syphilis is likely under-recognized, and as such, case-level data for undiagnosed patients is unknowable. By relying on reports from CPSP respondents, the gathered data will likely under-represent certain populations of children (e.g., children in rural areas, or children with limited access to paediatricians and paediatric subspecialists). Data elements not collected during routine care, including important points of history, physical exam, and diagnostic workup, will be absent from surveillance results. Longitudinal case-level data, which would be of academic interest, will also not be collected. However, the CPSP surveillance vehicle will be sufficient in addressing the study’s primary objectives with the above limitations taken into consideration.

**Ethics approval**

- Health Canada and Public Health Agency of Canada Research Ethics Board

### Analysis and publication

Interim analysis will be performed at the 6, 12, and 18-month marks of the study using descriptive statistics. Proportions will be analyzed using chi square tests and t-tests. Final analysis of gathered data will be completed within six months of the study’s closure.

In addition to the deliverables for multi-year studies, presentations will be made at annual international conferences. Publication in a high-impact journal, such as the *Journal of Infectious Diseases*, *Pediatrics*, or one of many public health or microbiology journals, is anticipated. Furthermore, the information gathered in this surveillance project may inform a new Canadian Paediatric Society position statement. Given the considerable lay-media attention around the “rise of syphilis,” a media article may be an effective way of sharing findings with the public. If there is local variability in the national distribution of congenital syphilis or its management, study findings may inform a targeted intervention to prevent future cases.

**References**

1. Expert Working Group for the Canadian Guidelines on Sexually Transmitted Infections. Syphilis. In: Wong T, Latham-Carmanico C, editors. *Canadian Guidelines on Sexually Transmitted Infections*, 2010. Ottawa, ON: Public Health Agency of Canada; 2010. https://www.canada.ca/en/public-health/services/infectious-diseases/sexual-health-sexually-transmitted-infections/canadian-guidelines.html
2. Syphilis rates in province highest since 1948, Alberta Health warns. CBC News [Internet]. 2019 Jul 16. [cited 2019 Sep 16]. Available from: https://www.cbc.ca/news/canada/edmonton/syphilis-alberta-health-outbreak-warning-1.5213851
3. American Academy of Pediatrics. Syphilis. In: Kimberlin DW, Brady MT, Jackson MA, Long SS, editors. *Redbook: 2018 Report of the Committee on Infectious Diseases*. 31^st^ ed. Itasca, IL: American Academy of Pediatrics; 2018:595
4. Public Health Agency of Canada. Reported cases from 1924 to 2018 in Canada - Notifiable diseases online: http://diseases.canada.ca/notifiable/charts?c= (Accessed November 18, 2020)
5. Dr. Jared Bullard, e-mail message to author containing unpublished public health data, September 16, 2019
6. Gleason C, Devaskar SU. *Avery’s Diseases of the Newborn.* 9th ed. 2012

**Detailed questionnaire:**

**Congenital syphilis**

| **REPORTING INFORMATION**  (To be completed by the CPSP) | |
| --- | --- |
| Report number: |  |
| Month of reporting: |  |
| Province: |  |
| Today’s date: |  |

**CANADIAN PAEDIATRIC SURVEILLANCE PROGRAM**

100-2305 St. Laurent Blvd.

Ottawa, ON K1G 4J8

Tel: 613-526-9397, ext. 239

Fax: 613-526-3332

cpsp@cps.ca

www.cpsp.cps.ca

**Please complete the following sections for the case identified above. If the information asked for below is not readily available, please leave it blank. Strict confidentiality of information will be assured.**

**CASE DEFINITION FOR CONGENITAL SYPHILIS**

Report any neonate, infant, or child <4 years old with a new diagnosis of confirmed or probable congenital syphilis in the last month.

**Confirmed congenital syphilis (requires one of the following)**

1. Identification of *Treponema pallidum* in the infant/child’s specimen by polymerase chain reaction (PCR) or fluorescent antibody examination
2. Reactive serology from venous blood in an infant that is four-fold greater than the maternal serology collected near the time of birth
3. Reactive serology from venous blood in an infant that persists beyond their second birthday

**Probable congenital syphilis**

1.	Infant born to a mother who had untreated or inadequately treated syphilis at delivery, regardless of findings in the infant

OR BOTH OF THE FOLLOWING:

2.	An infant or child with a reactive treponemal test result

3.	One of the following additional criteria:

a. Clinical signs of congenital syphilis on physical examination

b. Evidence of congenital syphilis on radiographs of long bones

c. Abnormal cerebrospinal fluid cell count or protein without other cause

d. Reactive treponemal immunoglobulin M (IgM) (19S-IgM antibody test or IgM enzyme-linked immunosorbent assay)

**Please specify if the case you are reporting is**: 🔾 Confirmed congenital syphilis 🔾 Probable congenital syphilis

**SECTION 1 – PROVIDER**

1.1 Do you have access to an infectious diseases specialist (by telephone or in person)? 🔾 Yes	🔾 No	🔾 I am one

**SECTION 2 – PATIENT DEMOGRAPHIC INFORMATION**

2.1 Month and year of birth: _____ /_______	2.2 Sex: 🔾 Male	🔾 Female	🔾 Intersex

MM	YYYY

2.3 Province/territory of birth: _______________	2.4 First 3 digits of postal code: ____	____	____

**SECTION 3 – MATERNAL AND PRENATAL HISTORY**

3.1 Maternal age at time of delivery: 🔾____ years	🔾 Unknown

3.2 Maternal population group (select all that apply):

| ❑ Arab | ❑ Black | ❑ Chinese | ❑ Filipino |
| --- | --- | --- | --- |
| ❑ Japanese | ❑ Korean | ❑ Latin American | ❑ White |
| ❑ First Nations | ❑ Inuit | ❑ Métis | ❑ Unknown |
| ❑ Southeast Asian  (e.g., Vietnamese, Cambodian, Laotian) | ❑ South Asian  (e.g., East Indian, Pakistani, Sri Lankan) | ❑ West Asian  (e.g., Iranian, Afghan) | ❑ Other, specify: _________________ |

3.3 Has the mother lived in Canada for less than 10 years? 🔾 Yes	🔾 No	🔾 Unknown

3.4 **Maternal social/behavioural characteristics:**

| Substance use in pregnancy? | 🔾 Yes | 🔾 No | 🔾 Unknown |
| --- | --- | --- | --- |
| Specify (if known): ______________________ | | | |
| Injection drug use? | 🔾 Yes | 🔾 No | 🔾 Unknown |
| Housing insecurity/homelessness? | 🔾 Yes | 🔾 No | 🔾 Unknown |
| Sex work/trafficking? | 🔾 Yes | 🔾 No | 🔾 Unknown |
| Involvement of child protection with other children? | 🔾 Yes | 🔾 No | 🔾 Unknown |
| Receiving social welfare assistance? | 🔾 Yes | 🔾 No | 🔾 Unknown |

3.5 For each trimester of pregnancy, was at least one prenatal visit conducted?

| 🔾 Yes | 🔾 Only second and third trimester visits |  |
| --- | --- | --- |
| 🔾 Only third trimester visits | 🔾 No documented prenatal care | 🔾 Unknown |

3.6 What, if any, barriers to prenatal care did the mother experience?_______________________________________

___________________________________________________________________________________________

3.7 Was the mother screened for syphilis during pregnancy?	🔾 Yes	🔾 No	🔾 Unknown

***If no*, proceed to question 3.10.**

*If yes,* was the syphilis screen positive?

🔾 Yes; Initial rapid plasma regain (RPR) 1: _____	🔾 Yes; Initial RPR Unknown	🔾 No	🔾 Unknown

3.8	Maternal syphilis stage:

| 🔾 Primary (chancre, <3 weeks since infection) |  |  |  |
| --- | --- | --- | --- |
| 🔾 Secondary (rash, systemic symptoms) |  |  |  |
| 🔾 Latent (asymptomatic): Specify estimated date of infection: | ❑ <12 months ago | ❑ >12 months ago | ❑ Unknown |
| 🔾Tertiary (cardiovascular manifestations, neurosyphilis) | 🔾 Unknown |  |  |

3.9 Did the mother receive treatment for syphilis in pregnancy?	🔾 Yes	🔾 No	🔾 Unknown

***If no*, proceed to question 3.10.**

3.9.1 Antibiotics choice: 🔾 Benzathine penicillin G	🔾Other, specify: ________________	🔾 Unknown

3.9.2 Trimester of treatment (select all that apply):

❑ First	❑ Second	❑ Third	❑ <4 weeks before delivery only	❑ Unknown

3.9.3 Post-treatment RPR (fill in all known):

| _____ months after treatment | 🔾 Known; 1:_____ | 🔾 Unknown | 🔾 Not completed |
| --- | --- | --- | --- |
| _____ months after treatment | 🔾 Known; 1:_____ | 🔾 Unknown | 🔾 Not completed |
| _____ months after treatment | 🔾 Known; 1:_____ | 🔾 Unknown | 🔾 Not completed |

3.9.4 Interpretation: 🔾 Adequate response	🔾 Inadequate response	🔾 Unknown

3.10 Was the mother tested at/near the time of birth? 🔾 Yes	🔾 No	🔾 Unknown

*If yes*, specify: RPR 1: _____	🔾 Unknown

3.11 Maternal co-infections at any time during this pregnancy (select all that apply):

| ❑ Human immunodeficiency virus | ❑ Hepatitis B | ❑ Hepatitis C | ❑ Herpes simplex virus |
| --- | --- | --- | --- |
| ❑ Chlamydia | ❑ Gonorrhoea | ❑ No known co-infections | |
| ❑ Other, specify: __________ |  |  | |

**SECTION 4 – DIAGNOSIS AND EVALUATION OF PATIENT**

4.1	Month and year of diagnosis: _____ /_______

MM	YYYY

4.2 Was the patient screened for congenital syphilis at birth? 🔾 Yes	🔾 No	🔾 Unknown

*If yes*, patient’s RPR at birth: 🔾 Positive; 1: ____	🔾 Positive; result unknown	🔾 Negative	🔾 Unknown

4.3	How was the diagnosis of confirmed or probable congenital syphilis supported (select all that apply)?

**Physical examination findings:**

| ❑ Rash | ❑ Jaundice | ❑ Hepatomegaly | ❑ Splenomegaly | ❑ “Snuffles” (copious nasal secretions) |
| --- | --- | --- | --- | --- |
| ❑ Small for gestational age | | ❑ Prematurity; gestational age at birth ___weeks ___days | | |
| ❑ Other: _______________ | | | | |

**Hematologic/biochemical abnormalities:**

❑ Complete blood count, specify: Hemoglobin _____	White blood cell count _____	Platelet count ______

❑ Liver enzymes, specify:

Alanine transaminase (ALT) ____ Aspartate transaminase (AST)____ Alkaline phosphatase (ALP) ____

❑ Other, specify: ________________

**Microbiologic work-up:**

❑ Treponemal testing (TP-EIA, TP-PA, etc.) ___________

❑ Cerebrospinal fluid cell count, specify: White blood cell count _____ Red blood cell count ______

❑ Cerebrospinal fluid chemistry, specify: Protein ____ Glucose ____

❑ Cerebrospinal fluid venereal disease research laboratory (VDRL) test______

❑ Other, specify: __________

**Radiologic work-up:**

| ❑ Neuroimaging | Result: ❑ Abnormal ❑ Normal |
| --- | --- |
| ❑ Long bone X-rays | Result: ❑ Abnormal ❑ Normal |
| ❑ Abdominal ultrasound | Result: ❑ Abnormal ❑ Normal |
| ❑ Other, specify:___________ | |
| Findings:____________________________________________________________________ | |

**Other work-ups:**

| ❑ Ophthalmologic exam | Result: ❑ Abnormal ❑ Normal |  |
| --- | --- | --- |
| ❑ Hearing screen | Result: ❑ Abnormal ❑ Normal |  |

❑ Other, specify: ____________

**SECTION 5 – PATIENT CLINICAL MANAGEMENT AND COMPLICATIONS**

5.1 Antibiotic management:

Age at treatment initiation: 🔾 <1 week old	🔾 1–4 weeks old	🔾 4 weeks–2 years old	🔾 >2 years old

a) Antibiotic selection: 🔾 Aqueous penicillin G	🔾 Benzathine penicillin G	🔾 Other, specify: _________ 🔾 Unknown

b) Dose, specify: _________________	🔾 units/kg	🔾 mg/kg	🔾 Unknown

c) Dosing frequency: 🔾 Once	🔾 Daily 🔾 Weekly	🔾 q2 weeks 🔾 q6 hours	🔾 q8 hours 🔾 q12 hours 🔾 Unknown

d) Duration of therapy, specify: ____________________ 🔾 Dose(s)	🔾 Days	🔾 Weeks	🔾 Unknown

5.2 Complications of confirmed or probable congenital syphilis (select all that apply):

| ❑ Death | ❑ Liver failure | | ❑ Coagulopathy |
| --- | --- | --- | --- |
| ❑ Seizures | ❑ Hydrops fetalis | | ❑ Encephalopathy |
| ❑ Neurosyphilis | ❑ Sensorineural hearing loss | | ❑ Perichondritis/osteochondritis |
| ❑ None of the above | ❑ Hospitalization in intensive care unit | ❑ Other, specify:______________ | |
| ❑ Unknown |  |  | |

🔾 **I agree to be contacted by the CPSP for further information on this questionnaire.**

🔾 **I do not wish to be contacted by the CPSP for further information on this questionnaire.**

**section 6 – REPORTING PHYSICIAN**

First name_____________________________	Surname_____________________________________________________________

Address_____________________________________________________________________________________________________

City___________________________________________	Province______________________________	Postal code___________

Telephone number___________________________________________	Fax number_____________________________________

E-mail______________________________________________________	Date completed_________________________________

**Thank you for completing this form**

**Supplement 3: Categorization of barriers to prenatal care**

Barriers to prenatal care were collected as free-text responses in the questionnaire. We reported the most frequent responses (substance use and housing issue) as their own category. Other low-frequency responses were categorized as structural or attitudinal barriers as shown in the table below. Some barriers may be associated; they were categorized as reported. For some M/BP, more than one response for barriers was reported and responses were categorized separately. References for the classification scheme used are provided in the footnotes.

| **Classification** | **Barriers** |
| --- | --- |
| **Structural barriers** | Not knowing where to get prenatal care |
|  | Having a long wait to get an appointment |
|  | Socioeconomically disadvantaged neighborhood |
|  | All nearby centers closed, limited prenatal care access |
|  | Northern or rural residence |
|  | Transportation |
| **M/BP attitudinal barriers** | Not planning or knowing about the pregnancy |
|  | Thinking of having an abortion |
|  | Believing they did not need prenatal care |
|  | Fear of losing other children |

References:

Heaman, M.I., Moffatt, M., Elliott, L. et al. (2014). Barriers, motivators and facilitators related to prenatal care utilization among inner-city women in Winnipeg, Canada: a case–control study. BMC Pregnancy Childbirth 14, 227. https://doi.org/10.1186/1471-2393-14-227

Heaman, M. I., Martens, P. J., Brownell, M. D., Chartier, M. J., Thiessen, K. R., Derksen, S. A., & Helewa, M. E. (2018). Inequities in utilization of prenatal care: a population-based study in the Canadian province of Manitoba. *BMC pregnancy and childbirth, 18*(1), 430. https://doi.org/10.1186/s12884-018-2061-1
